# Is medication administration in the elderly influenced by nurses’ demographics in South Africa?

**DOI:** 10.4102/hsag.v27i0.1750

**Published:** 2022-03-18

**Authors:** Emerentia C. Nicholson, Anneleen Damons

**Affiliations:** 1 Department of Nursing and Midwifery, Faculty of Medicine and Health Sciences, Stellenbosch University, Cape Town, South Africa

**Keywords:** elderly, medication administration, medication errors, medication, nurses, skill mix, staffing

## Abstract

**Background:**

Globally, nurses are increasingly employed post-retirement, with task-shifting to nurses with lower competencies, a lack of knowledge being a barrier, all of which could lead to medication errors.

**Aim:**

To describe the impact of nurses’ age, experience, training, and skill mix on the medication administration processes in long-term care facilities (LTCFs).

**Setting:**

Nurses (*N*=123) working in 28 LTCFs in the Western Cape province, South Africa.

**Methods:**

A quantitative non-experimental, cross-sectional descriptive design was used. The LTCFs were divided into funded (state-subsidised) and private (for profit) facilities using a stratified sampling method and each stratum thereafter randomised to obtain equal samples from each stratum. Self-administered questionnaires with close-ended statements were used, and statistical software (SPSS version 27) to perform descriptive and inferential analyses.

**Results:**

Respondents, (27%), had more than nine years of experience, with 15.8% aged 61-70 years; and 3.5% aged 71-80 years. Some were ‘very inexperienced’ in computer use (29.3%), 35% received medication training longer than five years ago, and *n*=28 nurses administered medication outside their scope of practice. The highest sources of job pressure were an increase in workloads (75.6%), being under stress (42.3%), and being overworked (39.0%).

**Conclusion:**

The aging nursing workforce, although experienced, found the job demands, paperwork, and technology barriers. Outdated training and delegating medication administration to lower categories of nurses can lead to medication errors.

**Contribution:**

This study’s findings can serve as a guideline for creating succession plans, recruiting procedures, development, and training of nurses, and improving clinical practices.

## Introduction

In South Africa, long-term care facilities (LTCFs) offer frail care to older persons. Furthermore, an older person is defined as a female aged 60 or more, or a male 65 years and older. These LTCFs provide 24-h frail care based on the older person’s physical or mental status (RSA 2006). The private for-profit, or the public sectors through non-governmental and faith-based organisations, deliver these services. Although the National Department of Social Development is the main department providing care to the elderly, intersectoral collaboration occurs between various other departments such as the National Department of Health.

When operating health systems, human resources are an essential input. However, the provision of staff entails having enough employees and the right mix of competencies. The term ‘nursing staffing levels’ refers to the total number of nurses providing the care or the ratio of nurses to residents. In contrast, the term ‘staff mix’ refers to the different categories of nurses providing the care (Backhaus et al. 2014). Various barriers still exist in supplying staff to healthcare services, such as skill-mix disparities, inadequate distribution of staff between services, gender imbalances and an aging workforce (WHO & Health Workforce Department 2008). To provide sufficient staff to care for the elderly, South Africa has a mandatory staffing model for LTCFs which is based on a 40-h workweek for the nursing and the support staff. This staffing model includes both the staffing levels and the staffing skills mix (RSA 2010). In the South African staffing model, the number of hours of care per week is calculated according to the level of care the residents need. Residents are categorised as follows: independent (no hours of care per week allocated), needs assistance (minimum care of 9 h per week required), and dependent on staff support (minimum care of 18 h per week needed) (RSA 2010). Despite a prescribed staffing model, an audit report on the quality of services in 405 LTCFs in South Africa indicated that in 2010, 21% of the LTCFs were still without access to professional registered nurses. This report stated that facilities supplemented nursing staff totals with lower levels of staff because of limited financial resources (Umhlaba Development Services 2010).

When determining the skill mix according to the prescribed staffing model, readers must note that the South African description of nurse categories differs from the categories recognised globally. Other countries describe the nurse categories as registered nurses, practical nurses, advanced practice nurses, licensed practical nurses and nurse associates (WHO 2017). In South Africa, there is a three-tier system comprising a professional and or registered nurse (RPN), registered staff nurse (RSN) and registered auxiliary nurse (RAN) (RSA 2005; SANC 2021a). In June 2019, the *Nursing Act* 33 of 2005 was updated to recognise the nurse categories as registered, general and enrolled nurses (ENs) (Department of Health 2019). Although the nomenclature of nurses was changed in the *Nursing Act* 33 of 2005 and legacy qualifications phased out, previous designations are still in use (RSA 2005). Reference in this article will thus be made to professional registered nurses (RNs), registered staff nurses as ENs, and registered auxiliary nurses/assistants (ENAs). These three categories of nurses provide the skilled component of nursing care, whilst caregivers offer further support. This study did not explore the role of the caregivers, because of their exclusion from medication administration tasks. The scope of practice for nurses in South Africa describes and differentiates between the professional roles and functions of the three categories of nurses (SANC 1984). The nurses’ scope of practice is currently under revision at the South African Nursing Council (SANC), although it is not finalised. A draft version inviting interested persons to submit comments was published on 12 May 2020, but the SANC extracted this version on 27 May 2020 as an incorrect version was released. On 03 July 2020, a revised version was published, again inviting interested persons to submit comments (Department of Health 2020a, 2020b; SANC 2021b). The scope of practice for South African nurses makes provision for the RNs to administer medicines and for the ENs to perform nursing care supervised by an RN, including the observation of reactions to medication. In contrast, the ENAs’ scope of practice does not provide for medication administration (SANC 1984). Despite this, the researchers’ prior knowledge that ENAs administered medication in some LTCFs in the Western Cape province (WCP) led to the inclusion of this nurse category in this study.

The researchers applied Donabedian’s Structure-Process-Outcome Quality of Care Model as a conceptual framework for this study (Donabedian 2005). According to Donabedian (2005), nurses are a structure or input measure that influences the practices associated with the standard of care (process measures). In turn, these practices that the nurses follow impact the outcome measures in terms of the care of patients (Donabedian 2005). In this study, the objectives were to determine the nurses’ age, experience and training, as well as the skill mix of the nurses working in the LTCFs and investigate the impact thereof on the medication administration processes in the LTCFs. This information could assist in creating succession plans, recruiting younger nurses, developing and training the nursing workforce, and improving clinical practices. This article is based on more extensive research that focussed on factors associated with safe medication administration in LTCFs.

## Methods

### Study design

The facilities were divided into funded (state-subsidised) and private (for profit) facilities using a stratified sampling method. Randomisation of each stratum followed, obtaining an equal sample of nurse respondents from the funded and private LTCFs. Data were collected via self-administered questionnaires.

### Setting

The study was conducted in 18 private and 10 funded LTCFs in the WCP, South Africa. Private facilities are for-profit and receive no government funding, whereas the government subsidises the funded facilities.

### Population and sampling

The original target population included all 430 nurses working in the 56 registered LTCFs in the WCP, South Africa. The 56 facilities were divided into two strata to include equal samples from each stratum. This led to *n* = 36 private and *n* = 20 funded facilities. After that, each stratum was randomised, and a 50% sample size was selected from each stratum, thus 18 private and 10 funded facilities. No further sampling was applied to incorporate all the nurses in the randomly chosen facilities. A total of *N* = 203 respondents were invited to participate, with *N* = 123 of the respondents returning the questionnaire, thus representing a response rate of 60.59%.

### Data collection

Professor Ala Szczepura from the Warwick Medical School, University of Warwick in the United Kingdom, permitted the use of the self-administered questionnaire (Szczepura, Wild & Nelson 2011). By pretesting the questionnaire on *N* = 17 (8.37%) respondents of the sample size of *N* = 203 within one private and one funded LTCF, errors were corrected to enhance the questionnaire’s appropriateness for the South African context, validity and reliability. These 17 respondents met the inclusion criteria, but their results were excluded from the main study. All the information conveyed within the questionnaire was self-reported by the respondents. The questionnaire included closed-ended questions with a choice of predefined statements, with the questions in dichotomous format and Likert scales. Sections included in the questionnaire were: socio-demographics, training, policies, medication administration, alterations to medication administration records (MARs), special circumstances in medication administration, job pressures and the use of computers and mobile devices. An online questionnaire was created by using an online application from Google. Data collection took place from 12 June and 30 August 2020 using both hard copies and online questionnaires.

### Data analysis

The data were analysed using the Statistical Package for the Social Sciences version 27 and a biostatistician’s assistance. Descriptive and inferential analyses were conducted. Statistical tests included: the Pearson product-moment correlation coefficient, the Pearson Chi-square, Spearman’s Rho 2-tailed statistical test, means and Cronbach’s Alpha.

### Ethical considerations

The Health Research Ethics Committee of Stellenbosch University approved this study (S19/10/252). Because of the COVID-19 pandemic and the implementation of a national lockdown, travel restrictions were in force (Department of Co-operative Governance 2020; Department of Co-operative Governance and Traditional Affairs 2020; RSA 2002). These restrictions led to the need to apply for a minor amendment to the original proposal (approved on 10 June 2020) to distribute both paper-based and online questionnaires. The researchers applied all the principles of research ethics, including informed consent from all respondents.

## Results

### Nurses’ demographic data

The respondents, *N* = 123 (100%) were as follows: RNs *n* = 60 (48.7%), ENs *n* = 35 (28.5%) and ENAs *n* = 28 (22.8%). All the respondents (100%) were female, and the LTCFs employed all. Of the respondents, 91.1% worked full-time shifts, with 8.9% working part-time for between two and four shifts per week. Of the *N* = 123, *n* = 114 (92.7%) provided their age, with 7.3% withholding this information. The mean age of the nurse respondents was 51.31 years. Their ages ranged from 22 to 77 years, with 39.5% in the age range between 51 and 60 years, thus nearing the retirement age. A total of 15.8% nurse respondents were between the retirement age of 61 and 70 years. Of the total RNs, 3.5% were in the post-retirement age group of 71–80 years (Table 1).

**TABLE 1:** Age distribution of respondents.

Age group	RNs (f%)	ENs (f%)	ENAs (f%)	Total *N* = (%)
21–30	0 (0.0)	3 (9.7)	2 (7.7)	5 (4.4)
31–40	4 (7.0)	5 (16.1)	4 (15.4)	13 (11.4)
41–50	9 (15.8)	12 (38.7)	8 (30.8)	29 (25.4)
51–60	24 (42.1)	11 (35.5)	10 (38.4)	45 (39.5)
61–70	16 (28.1)	0 (0.0)	2 (7.7)	18 (15.8)
71–80	4 (7.0)	0 (0.0)	0 (0.0)	4 (3.5)

**Total = *N***	**57**	**31**	**26**	***N* = 114**

RNs, registered nurses; ENs, enrolled nurses; ENAs, auxiliary nurses/assistants; f, frequency.

Thirty-four per cent of the respondents had four to nine years of work experience in LTCFs, with 27.6% having more than nine years of experience and 38.2% having less than four years of work experience in LTCFs (Table 2). Relative to the group sizes, the ENAs had the most experience working in LTCFs, as 46.4% indicated that they had worked longer than nine years in LTCFs: this compared with the ENs, 20%, and the RNs, 23.3% with more than nine years of experience in LTCFs.

**TABLE 2:** Nurses’ work experience in long-term care facilities.

Work experience	RNs (f%)	ENs (f%)	ENAs (f%)	Total *N* = (%)
1-12 months	3 (5.0)	3 (8.6)	1 (3.6)	7 (5.7)
> 1 year – ≤ 2 years	4 (6.7)	6 (17.1)	2 (7.1)	12 (9.8)
> 2 years – ≤ 3 years	4 (6.7)	3 (8.6)	1 (3.6)	8 (6.5)
> 3 years – ≤ 4 years	9 (15.0)	7 (20.0)	4 (14.3)	20 (16.3)
> 4 years – ≤ 9 years	26 (43.3)	9 (25.7)	7 (25.0)	42 (34.1)
> 9 years	14 (23.3)	7 (20.0)	13 (46.4)	34 (27.6)

**Total = *N***	**60**	**35**	**28**	**123 (100)**

RNs, registered nurses; ENs, enrolled nurses; ENAs, auxiliary nurses/assistants; f, frequency.

Respondents indicated their experience in computer use as ‘very inexperienced’, 29.3%, ‘fairly inexperienced’, 17.9%, ‘average’, 35.8%, ‘fairly experienced’, 13.0% and ‘very experienced’, 4.1%. Eighty-nine per cent of the ENAs never use a computer at work, in contrast to the ENs, 34.3% and RNs, 75.0%, who used a computer daily at work. The respondents primarily used computers for collating patient data and records, 43.1%, and work emails, 43.1%. The use of mobile phones at work was permitted for 83% of the RNs, compared with only 62.9% of ENs and 50% of ENAs. Respondents seldom used their mobile phones for medication-related tasks, such as a calculator for dosage calculations, 27.6% and searching for work-related information, 14.6%. These mobile devices were mainly used for communication: texting work-related people, 64.2%, instant messaging, 49.6%, and making work-related calls, 43.9%.

In the case of the RNs, 88.3% received registration as a nurse by completing a diploma course, and 10.0% achieved a degree. In addition, one of the RNs, 1.6%, had a master’s degree, with no respondents having a doctoral degree. A total of 6.5% were busy with further nursing studies that could lead to registration at the SANC. Only 15.4% of the total sample of *N* = 123 received the mandatory 6-monthly in-service medication training prescribed by the Department of Health (2011). Nineteen per cent of the respondents self-reported that they received medication training during the previous year, whilst 30.1% received medication training between one and five years ago, and 35.0% received their last medication training over five years ago. In addition, some respondents self-reported that they did not receive training on the side effects of common medications (with one not completing the question): *n* = 32 (26.2%). Furthermore, 22.8% of the respondents self-reported that they did not receive training on what common medications do, and 68.3% did not receive training on pre-checks before medication administration. A list of predefined statements was posed to the respondents as common reasons for medication errors. A lack of training was selected by (relative to the group sizes): RNs, 11.7%; ENs, 28.6%; and ENAs, 32.1%. In addition to medication training, only 20.3% received formal training in computer use.

Respondents were asked if they knew the purpose of the drugs they administered. In response, the RNs self-reported that 70.0% always knew the purpose, whilst 30% sometimes knew the purpose. Of the ENs, 45.7% always knew, and 54.3% *sometimes* knew the purpose of the drugs they administered. In contrast, all the ENAs self-reported that they only *sometimes* knew the purpose of the drugs. In addition, 36.7% of the RNs self-reported poor or insufficient knowledge of the actions and side effects of medicines as a reason for medication errors. In comparison, only 28.6% of the ENs and 21.4% of the ENAs agreed with this predefined statement. The Spearman’s rho test indicated a strong correlation between the respondents’ last medication training and poor or insufficient knowledge of the action and side effects of medicines, *p* = 0.005.

### Job pressures

**FIGURE 1:**
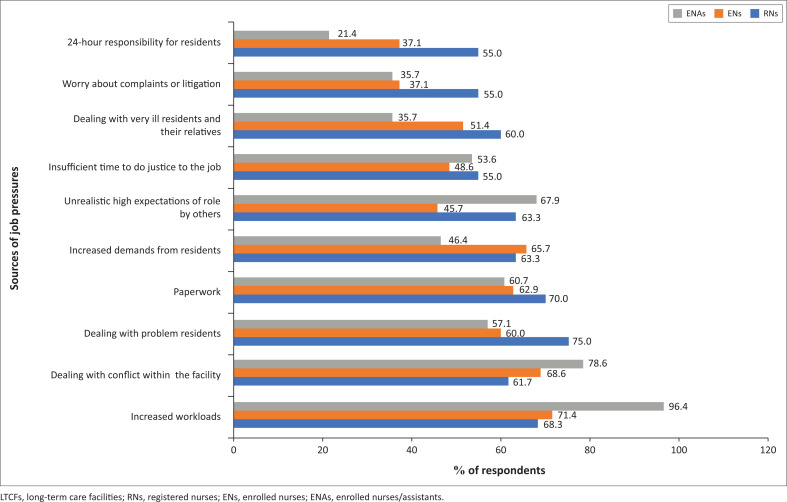
Top 10 sources of job pressures in the long-term care facilities.

The respondents were provided with 26 possible sources of job pressure and were then asked to rank these pressures ranging from 1 = no pressure, 2 = low pressure, 3 = moderate pressure and 4 = high pressure. Figure 1 summarises the results of the average of ‘moderate’ and ‘high pressure’ for the 10 highest job pressures; this is performed for each of the three nurse categories. The respondents identified the following: increased workloads, 75.6%; dealing with conflict within the LTCF, 67.5%; dealing with problem residents, 66.7%; paperwork, 65.9%; and increased demands from residents, 62.6%. In the case of the RNs, 75.0% found dealing with problem residents provided the most pressure. In comparison, the job pressure most experienced by 96.4% of the ENAs and 71.4% of the ENs was increased workloads.

### Processes of medication administration

Fifty-one per cent of the respondents self-reported that the most common method to dispense medication was a medication trolley taken directly to residents. Thirty-two per cent of the ENAs (in relation to the group sizes) only administered medication by bringing pill dispensers to the residents; these dispensers had already been pre-prepared in the nurses’ offices. A further 10.7% of the ENAs administered medication by bringing pre-prepared pill dispensers to residents combined with other dispensing methods such as taking a medication trolley to the residents. During the day shifts, the RNs and ENs administered most of the medication, but with increased use of the ENAs for administering medication during night shifts, in times of staff shortages and emergencies, 57.1%. Seventy-three per cent of the respondents self-reported that they perform drug rounds alone. For example, relative to the size of their group, 82% of the ENAs completed drug rounds independently. Respondents were asked to rank their comfort level with carrying out drug rounds alone from 1 = not at ease at all, 2 = somewhat uneasy, 3 = fairly at ease, and 4 = extremely at ease. Concerning the respondents’ comfort levels in performing this task, 91.7% of the RNs, 82.9% of the ENs and 85.7% of the ENAs self-reported that they were fairly to extremely at ease with carrying out drug rounds alone. The Spearman’s rank-order correlation test (*r* = 0.027, *p* = 0.765) showed no significant linear correlation between respondents’ years of work experience and how comfortable they felt performing drug rounds alone. A list of predefined statements was posed to the respondents as common reasons for errors in medication accountability. Respondents self-reported as follow: not signing for medication that was indeed administered, 56.9%; not recording reasons for non-administration, 56.1%; omission of times for ‘pro re nata’ (PRN) medications, 47.2%; no witness available to sign MAR changes, 35.0%; and not recording actual amounts, 27.6%. Again, a list of predefined statements was posed to the respondents, requiring them to select the most common resource-related reasons for medication errors. Respondents self-reported as follow: the staff are under stress, 42.3%; overworked staff, 39.0%; a shortage of appropriately qualified staff, 36.6%; staff was pressurised to complete drug rounds on time, 33.3%; poor or inadequate knowledge of the action and side effects of medications, 30.9%; and a lack of training, 21.1%.

## Discussion

Nurses in the LTCFs are human resources that influence the practices associated with the standard of care. Thus, the nurses’ processes will impact the outcomes in terms of resident care (Donabedian 2005). Therefore, it can be deduced that the nursing administration practices followed by the nurses can affect the health of the residents, such as making medication errors or not.

Sufficient human resources must be available to care for older people and correctly distributed according to their skills (Koopmans, Damen & Wagner 2018). Regarding staffing levels, Backhaus et al. (2014) suggested that there is no evidence showing that higher staffing levels necessarily lead to a better quality of care. However, when staffing levels are inadequate, such as in nursing homes with a nurse-to-resident ratio of 1:25 up to 1:36, nurses’ workload and stress levels increase (Al-Jumaili & Doucette 2018). Furthermore, increased workload levels are related to medication errors as nurses have many of other tasks to complete and can frequently be interrupted (Feleke, Mulatu & Yesmaw 2015). Seventy-five per cent of the respondents in this study also self-reported that increased workloads are the highest source of job pressure. They reported that the staff is pressurised to complete drug rounds on time, which was self-reported as a common resource-related reason for medication errors. Other resource-related reasons for medication errors in this study were that staffs are under stress, 42.3%, and overworked, 39.0%. Against this background, it should be observed that the World Health Organization (WHO) set the objective of developing policies that promote work conditions in healthcare settings. Moreover, improved work environments are conducive to quality care and the workforce’s motivation and can assist with the equal distribution of workforce totals and staff retention (WHO & Health Workforce Department 2008). Drennan and Ross (2019) also stated that a positive work environment facilitates the welfare of nurses, and by doing so, sustains and increases their motivation.

Job dissatisfaction was mentioned as the main reason for nursing staff leaving permanent employment, causing staff shortages. Job dissatisfaction is because of a lack of workplace resources, poor salaries, safety and poor career development opportunities. This leads to nursing staff migrating to other countries, moonlighting with nursing agencies or permanently leaving the profession (Tshitangano 2013). In addition, statistics indicate that nurses are mainly employed in hospitals (Australia 63%, United States 61%) and to a lesser extent in LTCFs (Australia 11%, United States 7%) (Drennan & Ross 2019). Locally, the SANC indicated that only 23% of ENs and 10% of ENAs were permanently employed in 2016, despite apparent nurse shortages (SANC 2016a). One strategy to address the nursing shortages is to retain and rehire nurses post-retirement. However, different countries have different retirement ages, whilst South African legislation does not prescribe any retirement age. Instead, the employer determines the retirement age, either in its employment contract or in a company policy document, which the employee agrees to accept upon signing the employment contract. For example, government employees can retire at age 55, 60 or 65 (Western Cape Government 2021). In 2017, the mean age of United States’ RNs was 51 years, with 13.7% being over 60 (Smiley et al. 2019). In South Africa, in 2020, 17% of the South African RNs were older than 60, with 3% being more aged than 69 years. In the case of ENs, 8% were older than 60, with none being older than 69. The corresponding figures for the ENAs were 7% older than 60 and none older than 69 (SANC 2021c). In this study, the RNs were a more mature group with 28.1% between 61 and 70 and 7.0% between 71 and 80.

Using older nurses post-retirement to provide care to older people has some advantages. Positive perceptions of older nurses include satisfaction upon delivering meaningful contributions and financial reimbursement (Kaewpan & Peltzer 2019). Other authors support this view: in other words, post-retirement nurses may be productive, but the physical and mental demands of nursing can be challenging (Uthaman, Chua & Ang 2016). In this study, 27% of the respondents self-reported more than 9 years of work experience. According to Feleke et al. (2015), nurses’ skills and knowledge of medication administration increase with work experience. With increased work experience, the nurses become more acquainted with different medication types and are least likely to commit medication errors (Feleke et al. 2015). However, the practice of using older nurses to provide care to older people also raises concerns. According to Kaewpan and Peltzer (2019), older nurses reported various barriers to working post-retirement. These included the job demands, diminishing physical abilities and increased paperwork. In the case of the older nursing workforce, such as in this study, it is also necessary to consider the risks arising from the current COVID-19 pandemic; thus, older adults have an increased risk of acquiring severe illnesses because of COVID-19. For example, for an adult between 65 and 74 years of age, the risk of hospitalisation because of COVID-19 is five times higher, and the risk of death is 90 times higher than for a person between 18 and 29 years (Centers for Disease Control and Prevention 2020).

The clinical staff over the age of 50 also have higher anxiety levels and poorer computer literacy skills than colleagues under 50 (Kuek & Hakkennes 2020). According to Kaewpan and Peltzer (2019), older nurses reported adjusting to new technology as a barrier to working post-retirement. An unexpected outcome of this study was the impact of the COVID-19 pandemic. The researchers were made aware by many respondents that they either lack the resources or the skills to complete online questionnaires. Therefore, of the 123 respondents, 52.0% completed paper-based questionnaires and 48.0% completed online questionnaires. Nurses need information technology skills because healthcare professionals increasingly use technology and devices such as computers, mobile devices and software applications. These skills are required for many applications, for example, accessing literature, identifying drug interactions, e-learning and monitoring patients’ health (Ventola 2014).

Furthermore, adequate training in information technology could lead to improved decision-making skills and better competencies; however, such training appears limited in the education of nurses (Topkaya & Kaya 2014). Also, bar code administration systems (BCMAs) are used to identify patients to ensure medication is administered to the right patient at the right time, giving the correct dose. A study suggests that 52% of residents were exposed, over 3 months, either to instances of attempting to administer medication to the wrong resident or to cases of administering discontinued medication (Szczepura et al. 2011). The findings of a systematic review examining the use of the BCMA in conjunction with other safety technology systems, for instance, a computerised prescribing and automated dispensing system, showed a reduction of 3.1% – 1.6% in potential adverse drug events (Shah et al. 2016).

According to Backhaus et al. (2014), a staff mix entails the different categories of nurses providing care. Lower-skilled nurses sometimes replace higher-skilled nurses to save costs (Aiken et al. 2017). Thus, task-shifting occurs, allocating tasks to health workers with lower skill levels and qualifications better to use the available human resources (WHO 2007). According to Aiken et al. (2017), when four RNs with two nurse assistants were on duty for 25 patients, the chances are that mortality would increase by 21% when substituting one RN post with a nurse assistant. Thus, lowering the nursing skill mix by increasing staff totals with lesser qualified and lower categories of nursing staff can decrease quality care and increase preventable deaths (Aiken et al. 2017). Other authors support the view that a staff mix consisting of higher qualified staff leads to a higher quality of care (Koopmans et al. 2018). In terms of the skill mix in this study, 67% of the ENAs selected from the list of the predefined answers to the question ‘shortage of appropriately qualified staff’ as grounds for medication errors, opposed to 34% of the ENs and 23% of the RNs.

The RNs in this study obtained their qualifications mainly through diplomas (88.3%), with 10.0% having bachelors’ degrees and one having a master’s degree. No respondents had a doctoral degree. This study’s findings align with literature in South Africa, which shows that graduate nurses are more likely to further their careers and be promoted to management positions or positions in education. This leaves nurses who have qualified with diplomas primarily to provide bedside care with limited involvement in scholarly activities. Consequently, less evidence is produced through research to advance evidence-based care (Roets, Botma & Grobler 2016). A small percentage of respondents (6.5%) were busy with further nursing studies. One part of the legacy nursing qualifications that are being phased out is the post-basic course in gerontology. Still, there is no substitute programme for nurses specialising in geriatric nursing (SANC 2016b).

Respondents in this study self-reported that their medication training was either outdated or lacking. This could impact the processes they follow during medication administration as this shortcoming was also self-reported by the respondents as a common reason for medication errors. In South Africa, the Department of Health prescribes mandatory 6-monthly medication training to nurses in LTCFs. Compliance with this mandatory training is monitored during health audits as part of the LTCF’s certification process (Department of Health 2011). Multiple studies identified the lack of knowledge on medication interaction, actions of medications and general pharmaceutical knowledge as potentially leading to medication errors (Al-Jumaili & Doucette 2017; Metsälä & Vaherkoski 2014; Szczepura et al. 2011). Also, a study conducted in Sweden, which examined 173 mandatory reports of serious adverse events, indicated that 64 reports were related to medication errors. Of these, 46 reports involved the staff’s lack of competence. In 33 instances of serious adverse events, assistant nurses administered the medication and these included 15 cases of the wrong dose or type of insulin (Andersson et al. 2018).

In South Africa, the administration of medication is a professional function assigned to RNs, although the ENs have the responsibility to observe the reaction of patients to medications. However, this observer responsibility falls outside the scope of practice of the ENAs (SANC 1984). Despite the limitations in the scope of practice for nurses, task-shifting does occur in the LTCFs. This task-shifting was evident in the 28 (100%) ENAs who partook in this study and reported administering medication to residents. The ENAs mainly administered medication by bringing pill dispensers to the residents; these dispensers had been pre-prepared in the nurses’ office. Eighty-two per cent of the ENAs performed drug rounds alone and were fair to extremely at ease with this process. This was even though 35% of respondents selected from the predefined list of answers to the question, the absence of witnesses to sign changes on the MARs was a common error of medication accountability.

## Limitations

The researchers did not explore why the ENAs are called upon to administer medication beyond their scope of practice. The researchers also compared responses by category of nurses, which could lead to social-desirability bias because of the cautiousness of the RNs to describe their concerns. Furthermore, the questionnaire relied on self-reporting by the nurses and not the actual observing of the medication administration processes. The close-ended questions in the survey instrument had predefined answers, which restricted the answers provided by the respondents. This could also have been a barrier to obtaining in-depth knowledge. Only one of South Africa’s nine provinces was included in the study, limiting the generalisability of the results. In addition, data collection was negatively influenced by the COVID-19 pandemic.

## Conclusion

The study explored the nurses’ age, experience and training, as well as the skill mix of the nurses working in the LTCFs and the impact thereof on medication administration processes in LTCFs. All information conveyed within the questionnaire was self-reported by the respondents and did not include the actual observing of medication administration processes. Quantitative data were collected from 123 nurse respondents in 28 LTCFs. The questionnaire contained close-ended questions in dichotomous format and Likert scales. Predefined statements were posed, which the respondents could select. The profile of the nurses in this study indicates an older workforce with experience in care for older persons. A positive impact of a more aging nursing workforce with work experience is that their skills and knowledge increase as their work experience increase. Consequently, they are less likely to make medication errors. On the negative side, older nurses experience diminishing physical abilities and find the job demands and paperwork barriers. Also, older nurses have an increased risk of acquiring severe illnesses because of COVID-19. Older nurses also find adjusting to new technology a barrier. Poor technology skills are a disadvantage when considering the increased use of technology such as BCMA to lower potential medication errors. The RNs in this study were mainly educated to diploma level. Research indicated that nurses who have qualified with diplomas primarily provide bedside care, so their involvement in research activities is limited. This results in a minor contribution to evidence-based care.

Long-term care facilities delegated professional tasks such as administering medications to lower categories of nurses, even though this was beyond their scope of practice. The ENAs self-reported that they completed drug rounds alone, which led to the absence of witnesses to confirm medication changes on MARs. The lowering of the nursing skill mix can decrease the quality of care and increase avoidable deaths. Outdated training or a lack thereof, as self-reported by the nurses, can lead to a lack of knowledge on the actions and interactions of medicines. This lack of pharmaceutical knowledge can potentially lead to medication errors. In addition, the respondents self-reported that increased workloads were the primary source of job pressure and that staff members were under stress. The increased workloads added pressure to staff to complete drug rounds on time, thus increasing the risk of medication errors.

The results from this study support the need for creating succession plans to obtain a balance between the experienced but aging workforce and recruiting younger nurses. Developing and training the nurse workforce can improve clinical practices. Future research should investigate the skill mix in LTCFs and how the delegation of medication administration to lower categories of nurses beyond their scope of practice can impact medication administration processes.
